# Recurrent osteoid osteoma of the index proximal phalanx: A case report with a new resection technique

**DOI:** 10.1097/MD.0000000000034490

**Published:** 2023-08-04

**Authors:** Joseph Antoine Mouawad, Mohamad Omar Youssef Honeine, Joe Georges Ghanimeh, Perla Naji Audi, Khalil Tanios Khalil

**Affiliations:** a Lebanese American University Medical Center-Rizk Hospital, LAU Gilbert and Rose-Marie Chaghoury School of Medicine, Beirut, Lebanon; b Lebanese American University Medical Center-St. John Hospital, LAU Gilbert and Rose-Marie Chaghoury School of Medicine, Beirut, Lebanon.

**Keywords:** case report, new surgical technique, osteoid osteoma, proximal phalanx, recurrence

## Abstract

Osteoid osteoma is a relatively common benign bone-forming lesion that often occurs in young adults with a predilection for the lower limbs and spine. It is characterized by the formation of osteoid tissue, a precursor to bone, and can cause pain and restricted mobility in affected areas. Osteoid osteoma of the hand and fingers is rare, thus representing a diagnostic challenge. A written informed consent was provided from the patient’s father for the publication and sharing all images and clinical data concerning the patient.

**Patient concerns:**

We are reporting a case of an 11-year-old girl who presented with persistent pain and swelling in her left index finger, as well as limited mobility, over the course of 1 year. Nonsteroidal anti-inflammatory drugs eased the pain, but it resurfaced once the medication was discontinued.

**Diagnoses:**

A series of investigations, showed a lytic lesion at the second proximal phalangeal neck, with features indicative of osteoid osteoma.

**Interventions:**

Excision with bone grafting was performed as definitive therapy with pathological confirmation of the osteoid osteoma diagnosis. Nevertheless, the patient returned 2 years later with a recurrence of her previous symptoms, and further tests suggested a recurrence of osteoid osteoma. To address the recurrence, a modified open thermoablation technique was used. Thermoablation is a minimally invasive procedure that uses heat to destroy the tumor cells, and it has been shown to be effective in treating osteoid osteoma. The modified open approach involves making a small incision to access the tumor and delivering heat directly through a previously CT-guided inserted Kirschner wire to the affected area.

**Outcomes:**

The patient reported no pain at 1 month and 1 year after the surgery, with no radiological signs of recurrence, indicating complete excision of the lesion.

**Lessons:**

Overall, this case highlights the challenges of diagnosing and treating osteoid osteoma in the hands and fingers. Further research is needed to better understand the underlying causes, potential risk factors, and optimal treatment for osteoid osteoma recurrence.

## 1. Introduction

Osteoid osteoma (OO), a common, benign, yet painful tumor, constitutes 3% of all bone tumors and 10% to 12% of benign bone tumors.^[1]^ It primarily affects men (male-to-female ratio, 2:1) and manifests in children and young adults.^[2]^ First described in 1935 by Jaffe HL, it is characterized by a reactive bone border surrounding an osteoid-rich core with varying degrees of calcification.^[3]^ The first and most frequently observed symptom of OO is localized pain associated with fusiform swelling. Pain typically worsens at night and is relieved by nonsteroidal anti-inflammatory drugs.^[4,5]^ It is radiologically described as a spherical single tumor, with a diameter of less than 1.5 cm, exhibiting a core zone of atypical bone known as the “nidus.”^[6]^ Although a computed tomography (CT) scan is thought to be the most precise imaging modality for diagnosing OO,^[5]^ magnetic resonance imaging (MRI) is considered more sensitive than a CT scan in phalanx OOs.^[2]^

The pathological features of OO are a small, round, soft, and hyperemic tumor surrounded by a harder and more sclerotic reactive bone. Microscopically, the nidus comprises a tissue of immature osteoid (woven bone) trabeculae with active osteoblastic rimming. Osteoclasts, capillaries, and nerve fibers can also be found.^[7,8]^

Approximately 50% of OO cases occur in the lower extremities (thigh and tibia), and only 5% to 10% occur in the hand and wrist.^[2,5]^ Involvement of the phalanx is a rare occurrence, with most instances documented in individual case reports. Delayed diagnosis and treatment are not uncommon because of the rare location and the unusual symptoms associated with it.^[9]^ Among these cases, a minority have displayed evidence of recurrence.^[5]^

**Figure 1.**
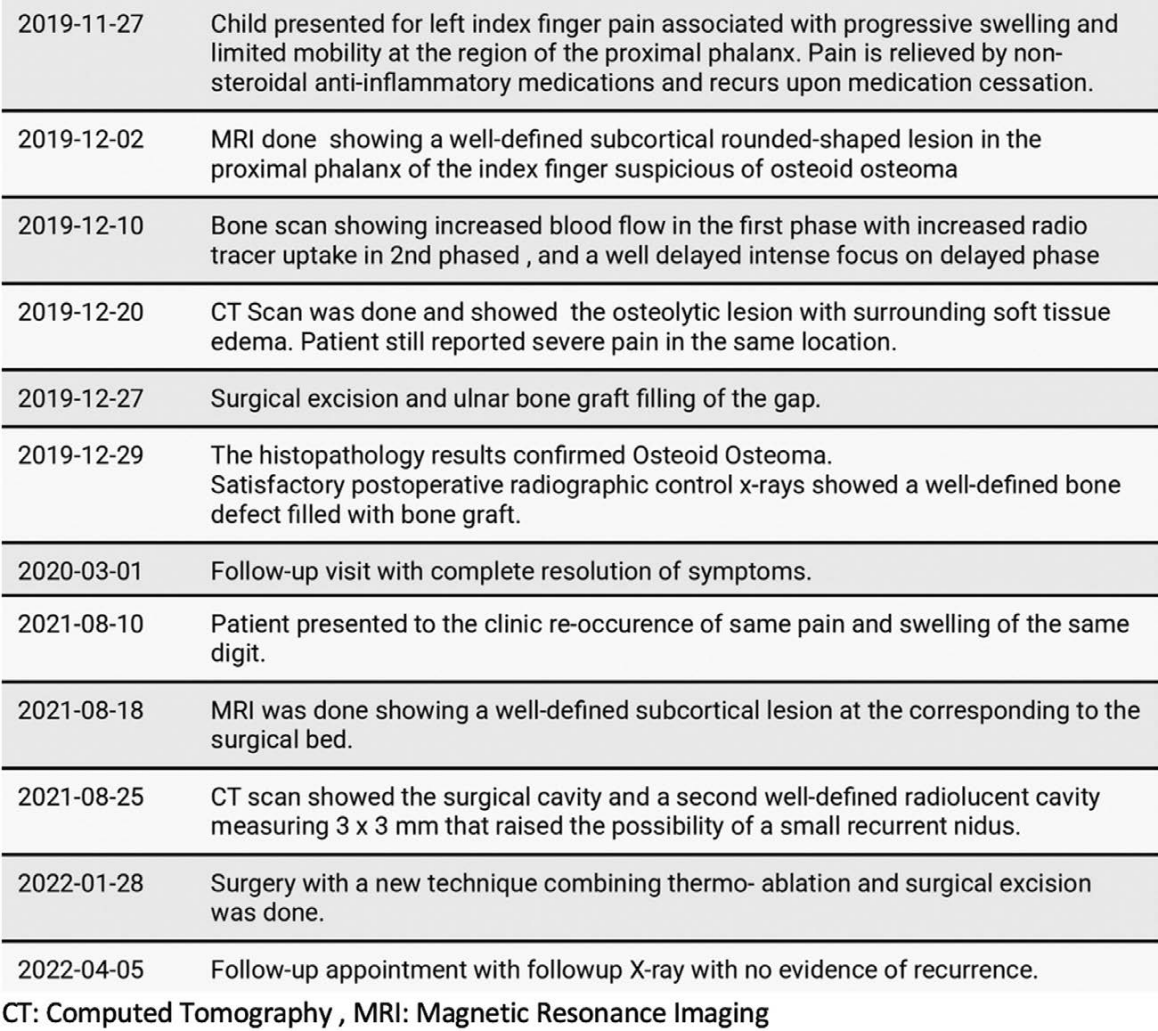
Case presentation timeline. CT = computed tomography, MRI = magnetic resonance imaging.

In this case report, we present the clinical course of a patient with OO located on the proximal phalanx of her second finger (Figure 1). Initial management involved surgical excision, but the lesion recurred subsequently. To address the recurrence, a novel modified open thermal ablation resection technique was employed, which resulted in complete resolution of symptoms and absence of residual disease on radiological imaging during follow-up. The case was managed at an academic private hospital, and the successful outcome of this modified technique highlights its potential usefulness in the management of recurrent OO.

## 2. Case presentation

Timeline of the case presentation is shown in Figure 1.

An 11-year-old girl presented with a 1-year history of left index finger pain associated with progressive swelling and limited mobility in the region of the proximal phalanx. She mentioned that her pain is relieved by nonsteroidal anti-inflammatory medications and recurs upon medication cessation. No previous trauma to the left index finger was mentioned. Upon physical examination, tenderness and swelling at the proximal phalanx of the index finger were noted, along with a range of motion limitation in the proximal interphalangeal joint.

**Figure 2.**
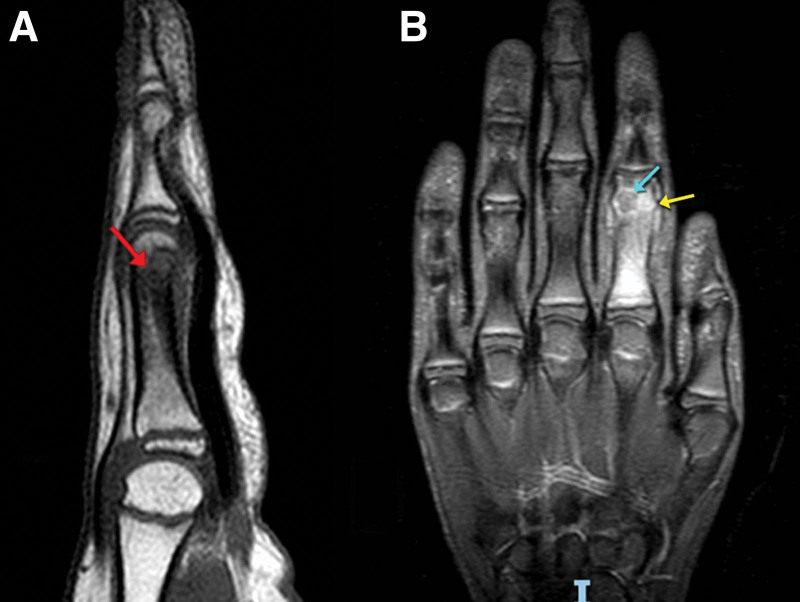
MRI done upon presentation showing the suspected lesion of the proximal phalanx of the index finger appearing hypointense on sagittal T1 WI (A) (red arrow), and slightly hyperintense on coronal T2 WI (B) (blue arrow) with surrounding tissue edema (yellow arrow). MRI = magnetic resonance imaging, WI = weighted imaging.

At that time, radiographs were taken and showed a lytic lesion localized in the proximal phalanx neck of the left index. An MRI was performed and showed extensive bone marrow edema at the level of the second proximal phalanx with evidence of a well-defined subcortical rounded-shaped lesion of 5 mm, appearing hypointense on T1-weighted imaging, slightly hyperintense on proton density, fat suppression, and T2-weighted imaging, raising suspicion for an OO with associated surrounding soft tissue edema (Fig. 2).

**Figure 3.**
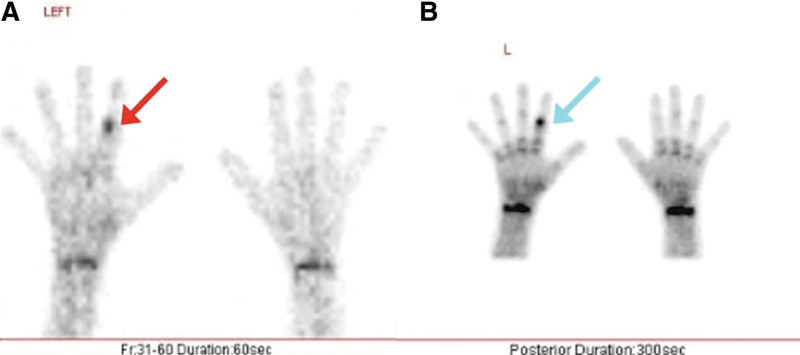
Bone scan showing increased blood flow in the first phase (A) (red arrow) and increased radiotracer uptake with a well-delineated focus (blue arrow) in the delayed phase (B) in the proximal phalanx of the left index finger.

Further investigation with a bone scan was done, showing increased metabolism in the region of interest. First-phase images showed focally increased blood flow to the region of the second left finger. Second-phase images showed increased radiotracer uptake in the soft tissues of the same area and abnormal early bone metabolism. Delayed imaging showed a well-delineated focus of intense uptake in the proximal phalanx of the left index finger. These findings were suggestive of an OO (Fig. 3).

**Figure 4.**
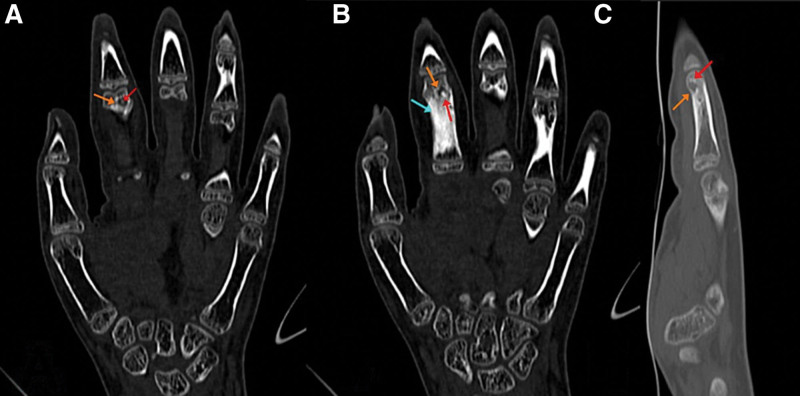
CT scan of the left hand with coronal (A and B) and sagittal (C) cuts showing a well-delineated, rounded shape, lytic lesion (orange arrows) with a central sclerotic dot (red arrows), and a sclerotic reaction of the shaft related to osseous hyperemia (blue arrow). CT = computed tomography.

**Figure 5.**
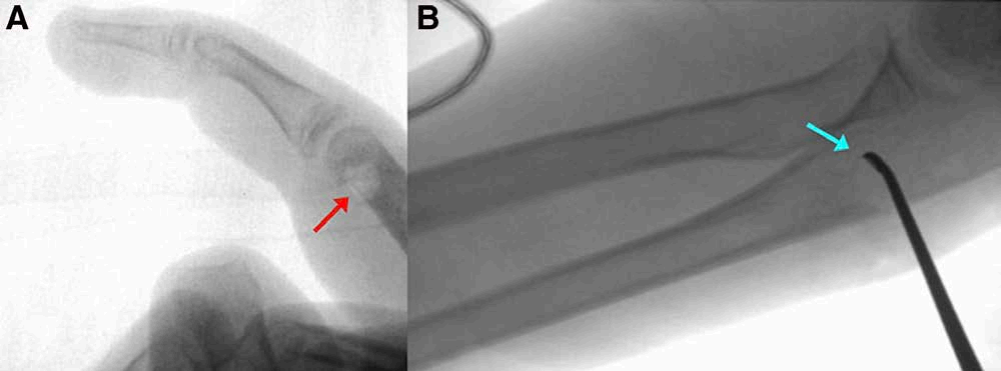
Intraoperative fluoroscopic views with coronal (A and B) and sagittal (C) cuts showing a well-delineated, rounded shape, lytic lesion (orange arrows) with a central sclerotic dot (red arrows), and a sclerotic reaction of the shaft related to osseous hyperemia (blue arrow).

A CT scan was also performed and demonstrated a well-defined subcortical lytic lesion at the second proximal phalangeal neck, with surrounding reactive sclerosis within the shaft suggestive of marrow hyperemia and surrounding soft tissue edema (Fig. 4). Those findings, though atypical given the absence of periosteal reaction, may be seen in the setting of an OO.^[10]^ A histopathologic correlation was then recommended for better evaluation and a definitive diagnosis. The patient was taken to the operating room for surgical excision and bone graft filling of the gap. Through an ulnar incision over the proximal phalanx head of the index finger, the cortex was identified and opened, curettage of the lesion was done, and samples were sent for histopathologic examination. The resulting gap was filled using a bone graft from the proximal ulna (Fig. 5).

**Figure 6.**
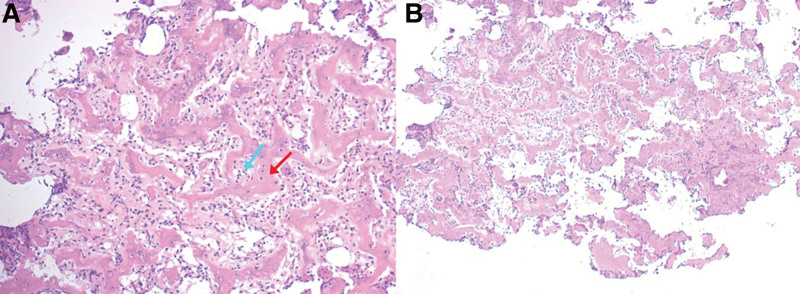
Histopathology Results showing an interlacing network of trabecula and osteoid bone (red arrow) with a central part (A) that is more mineralized than the peripheral part (B). A thin layer of osteoblasts (blue arrow) surrounded the trabecula.

The diagnosis of OO was confirmed by microscopic analysis of the tissue sample/sequestrum examined by a skilled pathologist. The histopathology revealed a network of interconnected trabecular and osteoid bone tissue with varying mineralization. It was observed that the core nidus was more mineralized than the surrounding area. A thin layer of tiny polygonal osteoblasts surrounded each bone trabecula (Fig. 6).

**Figure 7.**
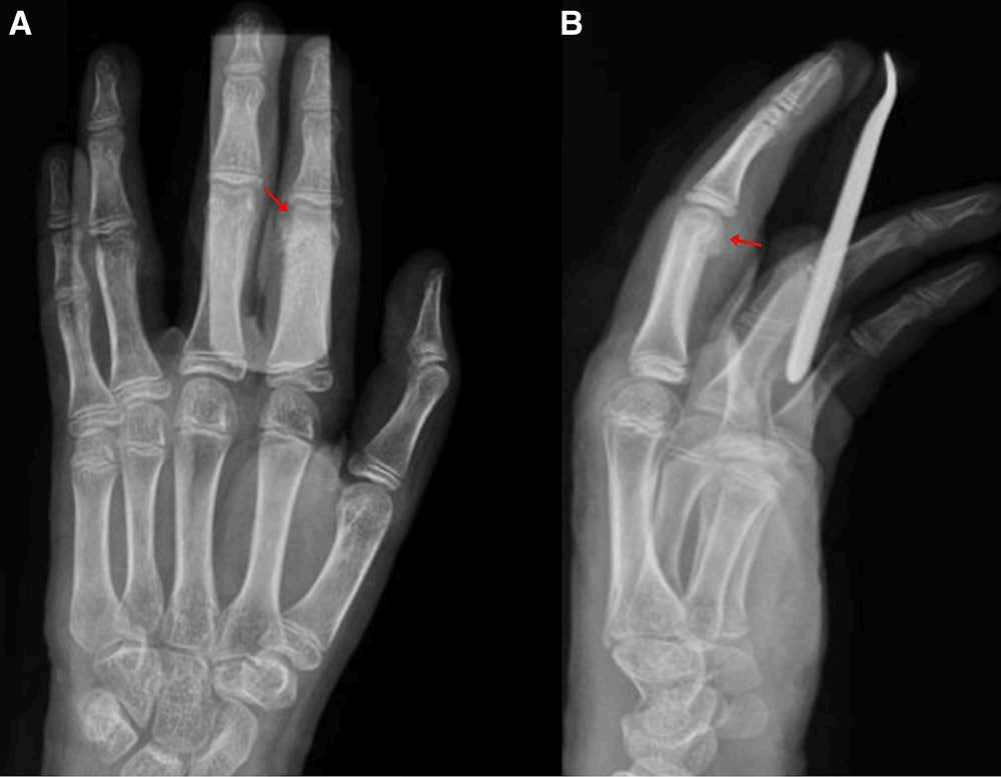
Postoperative X-ray images of the hand with anteroposterior (A) and lateral (B) postoperative radiographs of the hand with a volar splint and syndactyly taping showing a well-defined bone defect (red arrows).

Satisfactory postoperative radiographic control x-rays showed a well-defined bone defect at the second proximal phalangeal neck, almost filled with bone graft (Fig. 7).

The patient was followed up in the clinic 3 months postoperatively, with complete resolution of symptoms and full recovery of hand function.

**Figure 8.**
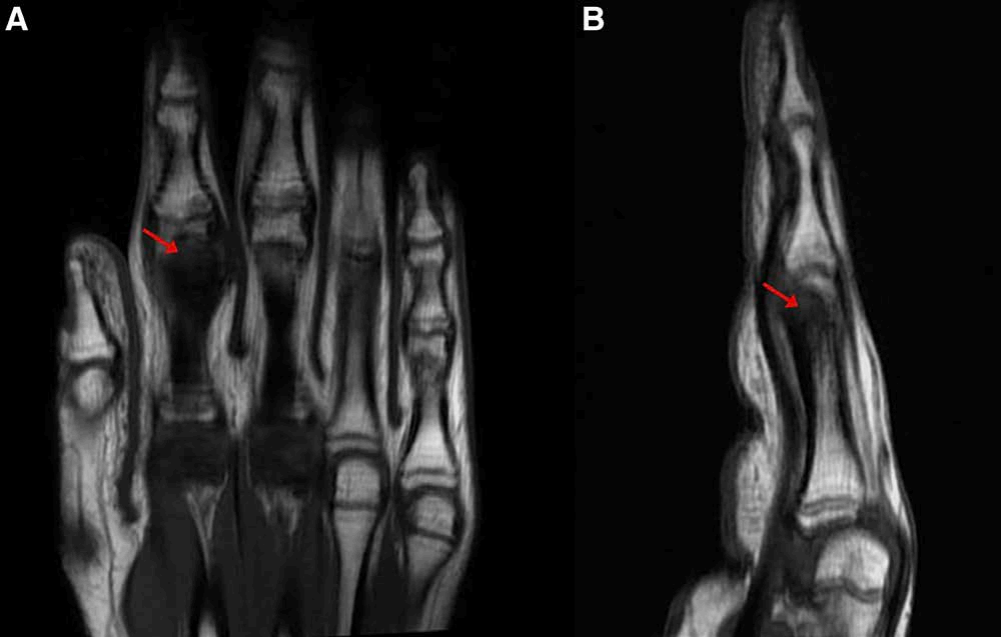
MRI T1 WI after reoccurrence of symptoms with coronal (A) and sagittal T1 (B) images showing the surgical cavity appearing as a hypointense lesion (red arrows). MRI = magnetic resonance imaging, WI = weighted imaging.

**Figure 9.**
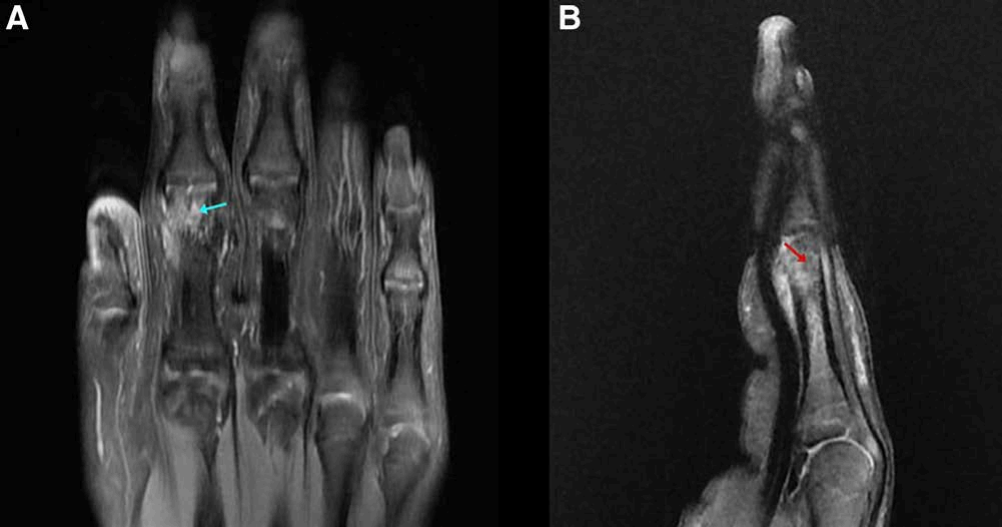
MRI FS cuts after reoccurrence of symptoms showing coronal (A) and sagittal (B) FS MRI cuts showing the surgical cavity (red arrow) with hyperintense surrounding soft tissue edema (blue arrow). FS = fat suppression, MRI = magnetic resonance imaging.

Two years after the procedure, the patient reeexperienced pain and swelling of the same digit with no history of trauma. Repeated radiographs and MRI showed a well-defined subcortical lesion at the ulnar and palmar aspects of the second proximal phalangeal neck, grossly measuring 6 × 5 × 10 mm, likely corresponding to the surgical bed. The lesion appeared hypointense on T1 and T2 weighted images, with surrounding marked bone marrow and soft tissue edema, which increased since the previous exam. Associated mild periosteal thickening and edema are also noted. Findings were highly suspicious for recurrent OO within the surgical bed (Figs. 8 and 9).

**Figure 10.**
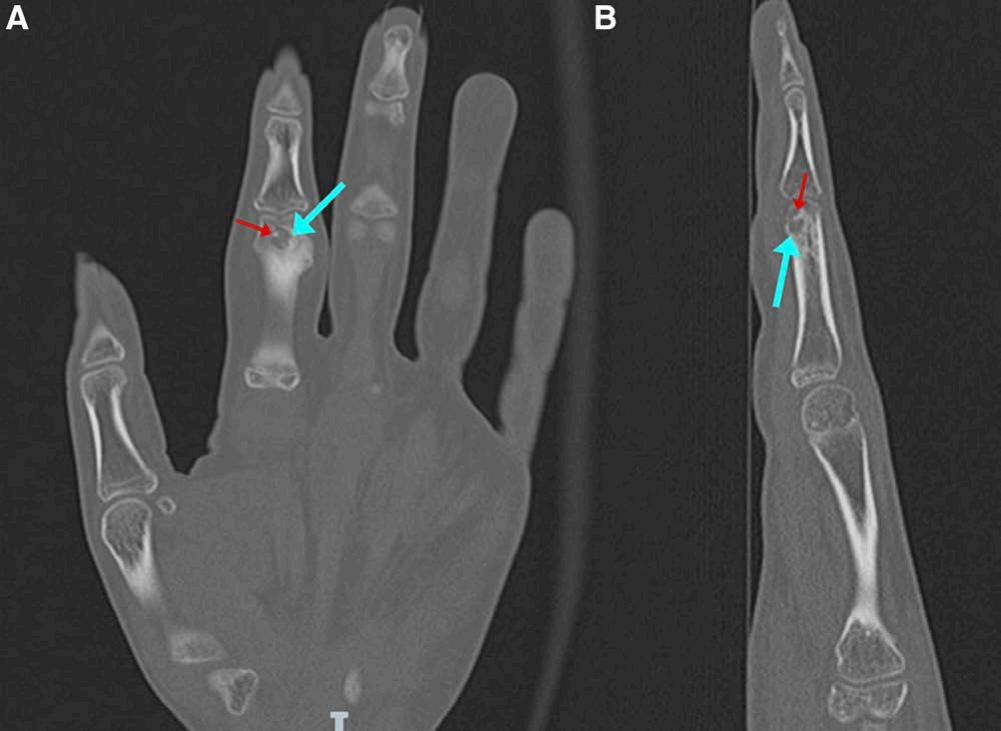
Coronal (A) and sagittal (B) CT scan cuts showing a well-defined surgical cavity (blue arrow) with a sclerotic center (red arrow). CT = computed tomography.

**Figure 11.**
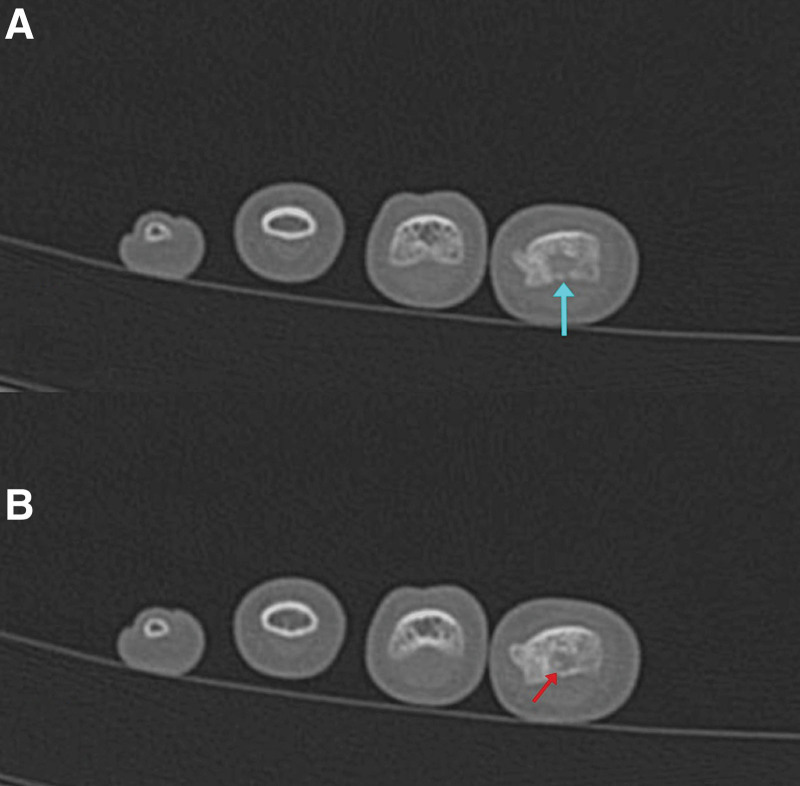
Axial CT scan cuts showing an osteolytic hypodense lesion (A) (blue arrow) with a sclerotic center (B) (red arrow) indicating recurrence. CT = computed tomography.

Subsequent assessment with a CT scan showed a 6 × 6 mm surgical cavity, associated with contiguous cortical and periosteal thickening, considered a sequela. Additionally, a second well-defined radiolucent cavity measuring 3 × 3 mm was identified, which contained a tiny central sclerotic dot that raised the possibility of a small recurrent nidus (Figs. 10 and 11).

**Figure 12.**
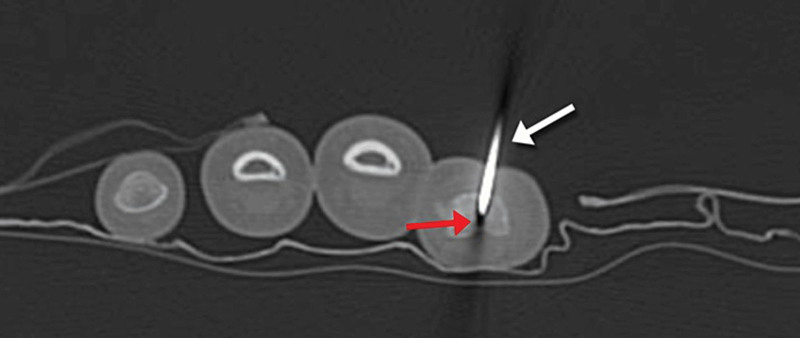
Axial CT scan cut showing the lesion (red arrow) tagged with a 1.2 mm K wire (white arrow) inserted under CT guidance. CT = computed tomography.

The decision was made to excise the mass using a new surgical technique that involved combining thermoablation and surgical excision. The patient was first taken to the CT room for lesion tagging, during which a 1.2 mm K-wire was inserted into the lesion nidus under CT guidance (Fig. 12).

The patient was then brought to the operating theater. Loco-regional anesthesia was performed. Scrubbing and draping of the left upper limb were done carefully. Using electrocautery, thermoablation of the lesion was realized by delivering heat into the tagged nidus through the previously inserted K-wire. Then, the surgical resection was performed through a longitudinal ulnar incision made over the old scar. Soft tissues were dissected and protected using retractors. The ulnar cortex of the proximal phalanx was opened using a motorized burr, and surgical excision of the lesion was pursued, targeting the nidus by reaching the tip of the K-wire. The patient tolerated the procedure well, with post-operative pain well controlled by analgesics.

The retrieved specimen was sent to the pathology lab. The pathology results were inconclusive, likely due to the suboptimal quality of the burned specimen. However, the fact that no definitive tumor cells were identified suggests that the electrocautery and burr ablation procedures were successful in eliminating any remaining tumoral tissue.

**Figure 13.**
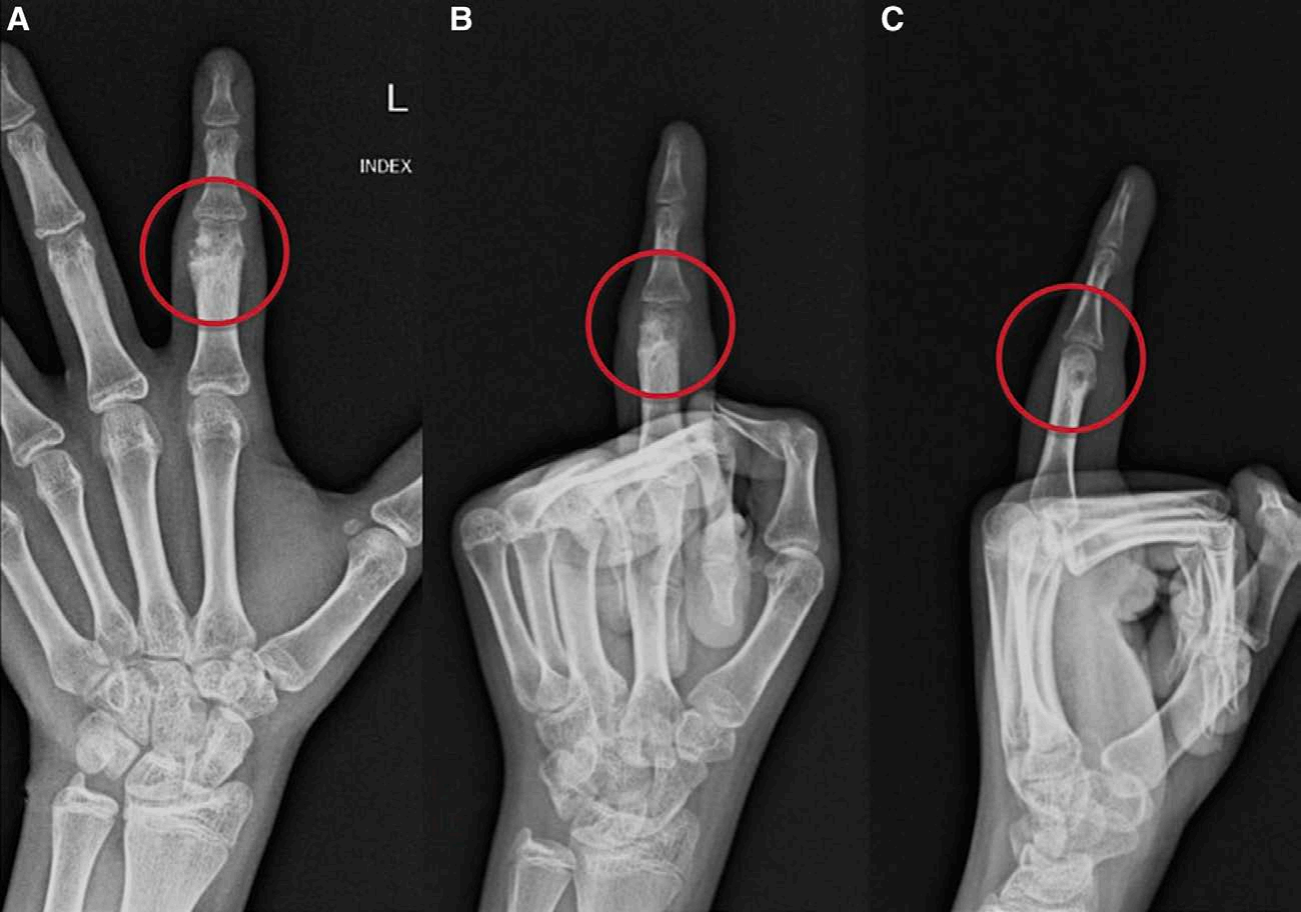
Control X-rays 1 year after the surgery showing anteroposterior (A), oblique (B), and lateral (C) radiographic views of the hand showing no evidence of recurrence of OO. OO = osteoid osteoma.

The patient underwent follow-up examinations 1 month and 1-year post-surgery, including control X-rays, which revealed no evidence of tumor recurrence (Fig. 13).

At the latest clinic appointment, the patient reported complete restoration of hand function. Although occasional soreness was noted at the surgical site, both clinical and radiographic assessments indicated no signs of tumor regrowth. Informed consent was obtained from the father of the patient for the publication of all images and case details since the patient belongs to the pediatric population.

## 3. Discussion

During the diagnosis and treatment of the present case, numerous challenges were encountered, with the first being the rarity of the lesion’s location. An extensive literature review conducted by Liu et al found only 171 cases of phalangeal OOs from 1985 to 2017, with only 7 cases localized in the first phalanx of the index finger, while the most common phalangeal site was the second phalanx of the middle finger with 34 occurrences. Additionally, OO seemed to affect the right hand nearly twice as much as the left hand.^[5]^

Although the vast majority of OOs (70%) typically develop in patients younger than 20 years,^[11]^ OO of the hand seems to affect older individuals, with an average age of 23 to 35 years upon diagnosis.^[5]^ Therefore, the age of our patient was another unusual feature.

Upon physical examination and primary X-ray imaging studies, the differential diagnosis included OO, enchondroma, and osteomyelitis, requiring further investigations. Recent literature guided the choice of MRI and scintigraphy, with MRI being a highly sensitive modality for hand OO, while scintigraphy has a detection rate of 96.5%.^[12]^

Conservative management with salicylates and nonsteroidal anti-inflammatory medications is an option, but the average time needed until the resolution of symptoms is 2 to 3 years.^[13]^ However, long-term nonsteroidal anti-inflammatory drugs lead to heavy GI disturbances, and some reports mention an increased probability of osteoblastoma transformation.^[14]^ Consequently, conservative treatment was excluded in our case.

Nearly one in 10 hand OOs treated surgically showed evidence of recurrence in the known literature.^[4]^ While no surgical technique has shown clear evidence of a reduced recurrence rate over another, curettage may increase this risk compared to en-bloc resection due to incomplete mass removal.^[5]^ This assumption led us to opt for an alternative technique when treating the patient’s recurrent lesion. Moreover, en-bloc resection was not considered due to the small bone stock and the high risk of fracture at this unusual location. Furthermore, a percutaneous radiofrequency ablation presented a high risk of soft tissue injury.^[15]^

Thus, the recurrence in our case was addressed using the combined thermoablation and surgical resection technique described previously in this article. Lanza et al^[16]^ reported a 5% recurrence rate for OO treated with percutaneous radiofrequency alone, the reason for which the combined excision technique was chosen by our team.

This case report sheds light on a rare instance of OO in the hand and fingers, providing valuable insights for clinical practice. It underscores the need to consider this diagnosis in patients experiencing finger pain and swelling, which can often be misdiagnosed. The report provides a detailed account of the diagnostic process, which includes imaging studies and histopathological examination, assisting clinicians in identifying OO in the hand and fingers. The report also describes a new, less invasive, modified open thermo-ablation technique for treating recurrent OO, which could be a useful alternative to traditional surgical excision. However, it is important to consider the limitations of the case report, including its single-case basis, the applicability of the treatment approach to all patients, and the lack of extensive documentation on long-term outcomes. Despite its limitations, this report offers valuable information on the diagnosis and management of OO in the hand and fingers, which can guide clinicians in improving patient care.

## 4. Patient perspective

Patient was very satisfied, pain free and has returned to daily life activities.

## 5. Conclusion

When a patient presents with an unusual mass on the phalanges of their hand, it is crucial to consider OO as part of the differential diagnosis. If conservative treatment methods prove ineffective or unsuitable, surgical options should be considered. The primary goal of the surgical treatment chosen must be to eliminate all tumoral tissue, particularly the nidus, in order to decrease the risk of recurrence. Thus, the combined thermoablation and surgical excision technique using CT-guided K-wire marking is a promising technique for the definitive treatment of OO.

## Acknowledgments

The authors would like to extend their sincerest gratitude to Dr Noha Bejjani for her willingness to collaborate and her openness to sharing her knowledge. Dr Bejjani’s expertise in pathology and her commitment to accurate diagnoses were critical to the success of this research. In addition, the authors also gratefully acknowledge the expertise of the interventional radiologist Dr Abdallah Noufaily for his valuable contributions to the successful application of the surgical technique described in this article. Prior permission has been obtained from all individuals mentioned in the acknowledgements section.

## Author contributions

**Formal analysis:** Perla Naji Audi, Joe Georges Ghanimeh, Khalil Tanios Khalil.

**Investigation:** Perla Naji Audi, Khalil Tanios Khalil.

**Methodology:** Joseph Antoine Mouawad, Khalil Tanios Khalil.

**Supervision:** Khalil Tanios Khalil.

**Writing – original draft:** Joseph Antoine Mouawad, Mohamad Omar Youssef Honeine, Joe Georges Ghanimeh.

**Writing – review & editing:** Joseph Antoine Mouawad, Mohamad Omar Youssef Honeine, Joe Georges Ghanimeh, Perla Naji Audi, Khalil Tanios Khalil.
